# Taking the First Steps Toward Integrating Testing and Training Cognitive Abilities Within High-Performance Athletes; Insights From a Professional German Football Club

**DOI:** 10.3389/fpsyg.2019.02773

**Published:** 2019-12-13

**Authors:** Adam Beavan, Jan Spielmann, Jan Mayer

**Affiliations:** ^1^TSG ResearchLab gGmbH, Zuzenhausen, Germany; ^2^TSG 1899 Hoffenheim, Zuzenhausen, Germany

**Keywords:** executive functions, game intelligence, computer testing, soccer, talent identification

## Introduction to Executive Functions

Executive functions (EFs) are higher-level cognitive functions which refer to the family of top-down mental processes that sub serve goal-directed behavior (Miller and Cohen, 2001), and are relevant in situations that require a fast and flexible adjustment of behavior to the changing demands of the environment (Zelazo et al., 2003). There is a general agreement that the three core EFs are: inhibition, working memory, and cognitive flexibility (Diamond, 2013). Previous research has proposed that EFs play an essential role in sport, connecting successful athletes with greater cognitive abilities (Jacobson and Matthaeus, 2014). Therefore, academics and practitioners alike are implementing EF testing batteries as an additional measure of performance. During the planning and implementation of EF assessments, there are obstacles that may be encountered by practitioners and coaches throughout all levels of play (i.e., amateur to professional leagues), such as the choice of assessments, the financial and opportunity costs, how to convey the data into meaningful results to the team, and what assumptions can currently be made from the data that is supported by research. By using the experience that we have gained by testing and training EFs for over 5 years at a professional 1st division football (Association Football) club in Germany, we aim to share our opinion on how to tackle these issues. We also aim to discuss the remaining barriers in EF research in hope of having more researchers and practitioners working together to collectively overcome them.

## Setting Up a Protocol

The choice of assessments is the foundation that all future assumptions are based upon, and it is recommended to have a test measuring each EF independently. However, a large hurdle that clubs will inevitably encounter is the financial cost of implementing new assessment tasks. Despite some companies marketing their cognitive testing for upwards of $30,000 (i.e., CANTAB), this does not mean that cognitive batteries should only be implemented by the teams with larger budgets. Assessments such as the Design Fluency task to assess cognitive flexibility, and the Digit Symbol Substitution task to assess working memory can both be completed by pen and paper, whereas the N-back task to assess working memory and the Stroop Task to assess response inhibition can be created using PowerPoint. Furthermore, establishing mutualistic relationships with universities can provide opportunities for teams to either use the psychological tools owned by the university in exchange of participants' data for research purposes, and/or to help develop their own EF assessments.

## Data Collection

Our club assesses every players' EFs once during the pre-season and once halfway through the season. We further recommend collecting contextual information about each player such as: birthdate, birth quartile and birthplace, intelligence quotient (IQ) or a similar academic grading score, history questionnaires on their years of experience playing both their main sport and any additional sports participation, and hours of training per week both in structured and unstructured playing environments (Mann et al., 2017). Contextual information can help improve our understanding of whether high-level athletes display better EFs than their lower-level and non-athletic counterparts because they were either born with greater cognitive abilities (i.e., nature), or whether their higher cognitive abilities are sport/environmentally-induced (i.e., nurture; Scharfen and Memmert, 2019).

## Communicating the Results

**Figure 1:**
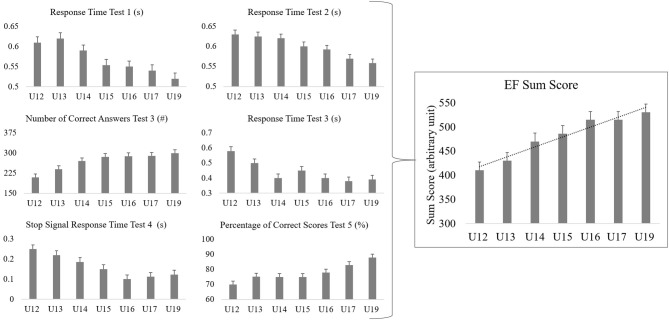
Mock data demonstrating how the results from individual assessments with various units can be combined in one summary graph. Values were normalized and then used in a two factor analyses to develop a total executive function sum score (EF sum score) for all players. A new EF variable was developed using each individual item's factor loading as a weighting system, so each change in one unit of performance was equal across each assessment. Our equation unique to our assessments (and serves only as an example) are: EF sum score = (0.720*Response Time Congruent Inverse) + (0.699*Response Time Incongruent Inverse) + (0.756*Determination Test Number of Correct Answers) + (0.828*Determination Test Response Time Inverse) + (0.853*SSRT Inverse) + (0.766*Response Inhibition Time Inverse).

Measuring EFs requires multiple complex assessments in a psychological domain and ensuring that the results are understood by the intended audience can be difficult (Eisenmann, 2017). Some strategies have been recommended in the literature. For example, Sakamoto et al. (2018) created a composite score by changing the results of each individual test into a *z*-score and then adding the z-scores together. In a practical sense, the idea of creating a single number that encompasses different scales for each variable can make the results easier to interpret and can be relatively easy to implement. However, caution should be taken in this approach as it may under-power the changes for each test. A more complex method that this club developed is an “EF sum score,” which combines all results into one total value (Beavan et al., 2019). Figure 1 displays the practicality of the sum score to provide a smoother translation of the relevant results to the intended audience (Buchheit, 2017).

## What Inferences Can Currently Be Made from EF Results?

We evaluate our players' EF performance relative to their age group norms. However, there is a high variation between players on EFs across each age group, and this has similarly been reported in another study (Sakamoto et al., 2018). Our practitioners report that players who are the true elite academy players that yield the potential to make it to the adult professional level are also the players who are outperforming their age-group in the EF assessments, and the variation is caused by the many athletes that do not yet hold this “elite” status amongst coaches. Longitudinal data is needed in order to support this standpoint, but an interesting next step is to relate EFs to a panel of coaches' ratings of skill and potential of each player to reach the professional level rather than their age. Furthermore, various levels of motivation may influence the attainment of scores across the battery, specifically in EF assessments where athletes may be disinterested in performing non-sport specific testing. Although this remains difficult to account for, the underlying principle is that practitioners should consider the subjective contextual variables when interpreting their team's EF results. Variables such as the presence of a coach, motivation, players' contract situations, wellness, and whether the participants understand why they are doing the assessments all may influence the test results (Stiroh, 2007); but these variables have remained difficult to account for objectively in scientific research that may help explain the observed variance.

Despite these obstacles, a recent meta-analysis reported that a positive finding exists regarding the importance of EFs in football (Scharfen and Memmert, 2019). Higher-level athletes demonstrated better EFs than lower level athletes, and it has therefore been advocated that EF testing could play a role within the talent identification process (Huijgen et al., 2015; Sakamoto et al., 2018; Montuori et al., 2019). Yet additional research outside of football did not confirm the generalization of better EFs linked with expertise, where no differences between different levels of expertise in tennis (Kida et al., 2005), ice hockey (Lundgren et al., 2016), or basketball (Nakamoto and Mori, 2008) were reported. Therefore, there is a lack of agreement in the literature on whether EFs as a prognostic tool for football talent has practical validity. Albeit, previous research has attempted to understand if EFs could help identify talent. For example, Sakamoto et al. (2018) reported that players who were accepted into an academy exhibited better EFs than players who were rejected. Yet we need to assess whether these statistically significant differences are also practically relevant. The between-group difference on a Stroop task was on average +3 correct answers out of 100 (rejected group: 31.3 ± 9.6; approved group: 34.5 ± 8.6; *p* = 0.001; effect size = 0.35). In other words, the groups that were accepted and not accepted into the academy overlapped by about 86% (Magnusson, 2014). Although the differences reached statistical significance, whether they are large enough to help a coach distinguish between a player that should be accepted or rejected from an academy remains questionable.

To date, longitudinal studies in athletic populations are lacking, leaving only weak generalizations of the EF developmental trajectories from existing longitudinal studies in general populations. Therefore, longitudinal research is needed to understand if assessments of EFs are able to help practitioners predict talent in young athletes. For example, does assessing EFs have more value to help in the detection of potential talent from a heterogenous cohort that does not yet compete at a high-level (i.e., a large group of school kids), or in the identification of the best performers within a homogenous cohort of already competing athletes (i.e., high-level academy players likely to become adult-professionals)? Currently, no study has yet to demonstrate that athletes with higher EF scores become more successful in their sport.

## Cognitive Training

The cognitive training approach stems from the “broad training hypothesis” which states that training basic cognitive skills could improve EFs and would therefore translate into better performances when utilizing EFs (Walton et al., 2018). For example, 10 sessions of cognitive training in a laboratory improved football players' on field passing decision-making accuracy by 15% (Romeas et al., 2016) and Ducrocq et al. (2016) reported the possibility to enhance sporting performance by improving the inhibitory control of tennis athletes. Importantly, there remains a large debate on whether training with computer-based cognitive tasks can broadly transfer into real-world performances. An extensive review by Simons et al. (2016) conveyed that no compelling evidence currently exists showing a true positive transfer of cognitive training interventions to real-world tasks. Recently, Harris et al. (2018) mentioned that although the lack of evidence across the literature is not an encouraging sign that it would work for athletes, only one study directly examined the benefits of a cognitive training program on sporting transfer task. Seemingly, if academics and practitioners are wanting to overcome this paucity of knowledge directly in sport, they are recommended to read Walton et al. (2018) who provides recommendations of how to best explore the link between cognitive and sporting abilities.

It is important that if clubs decide to invest in training EFs, the staff should further discuss both the financial cost of purchasing the equipment and the opportunity cost of spending the time and money on a different task (Simons et al., 2016). In order to reduce the opportunity cost, we emphasize the importance of cognitive training toward players who are: (i) regressing from their previous EF scores, (ii) wanting to engage in cognitive training, (iii) injured, and (iv) scoring in the lowest third within their age norms. The reason behind training players who performed in the lowest third is based on previous non-sporting literature reporting that a threshold effect may exist with natural abilities and expertise, where any improved ability beyond the requirement to compete at a high-level (i.e., the threshold) may not further improve performance (Terman and Oden, 1959). Contrastingly, this also means that players who are under the threshold may yield the potential to enhance their performance by improving their EFs (Diamond, 2016). Diamond (2016) advocated that training EFs are important to the future success of individuals and should begin as young as possible; especially in individuals that yield the lowest scores to ensure that their deficiencies are not enlarged over the coming years. Although the threshold hypothesis is still relatively new when explaining the role of EFs in sport, it has been used to explain differential correlations between intelligence (i.e., IQ), creativity (Jauk et al., 2013), and future career achievement (Terman and Oden, 1959; Baird, 1985; Gagné, 2004).

## Conclusion

Practitioners are commonly the first to apply and test new methods with science following in attempt to examine their practices. The measurement and training of cognitive abilities such as EFs are becoming a popular new approach in sporting clubs. However, with EF research being a relatively young area of research, the importance of EFs in sport remains widely unknown, and it remains unclear if the measurement and training of EFs are justifiable to help predict future talent. Pending further research, a current focus on EF development in the lower achieving athletes may be a more suitable use. Being well-informed of the scientific literature will help in overcoming the delicate balancing act between administering good scientific practice methodology and what is functional for the club with respect to the aforementioned hurdles of implementing the testing and training of EFs. Therefore, new research-practitioner relationships are a cornerstone to furthering our understanding of the role that EFs play in sport. Collectively, a promising opportunity exists to help overcome the limitations in the literature if research informed protocols are put in place with a purpose of improving the support toward the testing and training EFs.

## Author Contributions

AB, JS, and JM contributed toward the conception and ideas presented of the opinion piece. AB wrote the majority of the manuscript. JS wrote sections of the manuscript. All authors contributed to the manuscript revisions, read, and approved the submitted version.

### Conflict of Interest

The authors declare that the research was conducted in the absence of any commercial or financial relationships that could be construed as a potential conflict of interest.
